# Comparative Analysis of Oligosaccharide and Phenolic Profiles in White and Red Grape Pomace from California

**DOI:** 10.3390/molecules31142530

**Published:** 2026-07-21

**Authors:** Bruna Paviani, Xueqi Li, Han Peng, Mara Baller, Selina C. Wang, Daniela Barile

**Affiliations:** Department of Food Science and Technology, University of California, Davis, Davis, CA 95616, USA; bpaviani@ucdavis.edu (B.P.); spsli@ucdavis.edu (X.L.); hpeng@ucdavis.edu (H.P.); mara.baller@gmail.com (M.B.); scwang@ucdavis.edu (S.C.W.)

**Keywords:** prebiotics, gut health, upcycling, grape marc, LC-MS

## Abstract

The substantial volume of agrifood processing side streams represents a global sustainability challenge that requires transformation of agricultural residues into high-value molecular components. Grape pomace (GP) is a major winemaking co-product whose chemical diversity remains underutilized due to a lack of high-resolution structural data. This study applies advanced analytical platforms to provide a comprehensive molecular characterization of oligosaccharides (OS) and phenolics in GP from four grape varieties (Chardonnay, Sauvignon Blanc, Pinot Noir, and Merlot) from California. Results demonstrate that the molecular signature of the material is highly dependent on the variety and its corresponding processing; white wine pomaces exhibited significantly higher residual sugars and generally greater OS diversity compared to red wine pomaces. Using LC-Q-ToF-MS, 39 oligosaccharides were identified, primarily composed of hexoses and pentoses. Characterization of the OS building blocks via LC-QqQ-MS revealed the dominance of glucose and fructose, followed by arabinose and xylose. In parallel, targeted phenolics quantification by UPLC-DAD showed that (+)-catechin and (−)-epicatechin accounted for up to 50% of quantified phenolics in Chardonnay pomace and 45% in Pinot Noir pomace. This work highlights the molecular intricacies of GP, providing a compositional foundation for its targeted valorization.

## 1. Introduction

The United States ranks as the world’s fourth-largest wine producer, with California responsible for approximately 80% of total national wine production. Over the past decade, California has crushed roughly 4.5 million tons of grapes per year, with wine grapes accounting for ~95% of the crush [1]. During the winemaking process, ~30% of crushed grapes remain as a solid residue after pressing [2,3]. This material, commonly referred to in the literature as grape pomace (GP) or grape marc [4,5], includes grape skins (60%), seeds (35%), and stems (5%). Despite its large volume, GP is typically restricted to low-economic applications such as composting or biofuel generation [6,7]. This conventional management of agricultural co-products often overlooks their true economic potential as a source of high-value ingredients.

The chemical composition of GP is highly heterogeneous, influenced by the variety, environmental conditions and processing method [8]. While known to contain soluble sugars, polysaccharides and secondary metabolites, the industry often underestimates this diversity by treating GP as a uniform byproduct. Previous studies have reported soluble carbohydrate contents ranging from approximately 40 to 78% of dry matter in white GP, whereas values reported for red GP vary from approximately 1 to 15% of dry matter, depending on the pomace and analytical method used [9,10]. Conversely, red GP exhibits a significantly higher content of insoluble carbohydrates (51.1–56.3% *w*/*w*), whereas white GP has approximately half (17.3–28% *w*/*w*) [9,11]. These variations suggest that a generalized, non-specific approach to co-product utilization minimizes the inherent value of the biomass.

To reveal the true economic potential of these side streams, it is critical to apply advanced analytical techniques to map their molecular intricacies. Historically, GP’s bioactivity has been attributed primarily to phenolic antioxidants [12,13,14], largely neglecting the complex carbohydrate fraction. Emerging research into plant-derived oligosaccharides (OS) suggests that they represent a significant yet underutilized resource, as some exhibit prebiotic activity by supporting the growth of health-associated gut bacteria [15,16]. These non-digestible carbohydrates, typically exhibiting a degree of polymerization (DP) of 3 to 20, possess structural complexities (linkages and monosaccharide composition) that determine their functional efficiency in bioprocessing and nutrition [15]. Similarly, the transport and utility of GP phenolics are dictated by specific molecular weights and configurations [17,18]. Therefore, precise structural elucidation and quantification are the necessary precursors to any valorization strategy.

The lack of high-resolution variety-specific data remains a bottleneck for the circular bioeconomy. This study addresses that gap by applying high-resolution analytical platforms to GP from four major California varieties (Chardonnay, Sauvignon Blanc, Pinot Noir, Merlot). By evaluating multiple varieties, this research demonstrates that the molecular signature of the residue is a function of the specific variety and its corresponding processing. Such data are essential for feeding emerging global food composition platforms, such as the Periodic Table of Food Initiative (PTFI) [19] and other specialized platforms, like The ByProductDatabase, where this data will be incorporated (https://www.byproductdatabase.com/). These initiatives aim to standardize the chemical fingerprinting of the global food supply and its byproducts to optimize resource recovery [20]. Ultimately, this work seeks to demonstrate that analytical chemistry is not a merely descriptive tool, but a value-revealing mechanism essential for transitioning from conventional agricultural management to precision valorization.

## 2. Results

### 2.1. Soluble Sugars and Estimation of Oligosaccharides

**Figure 1:**
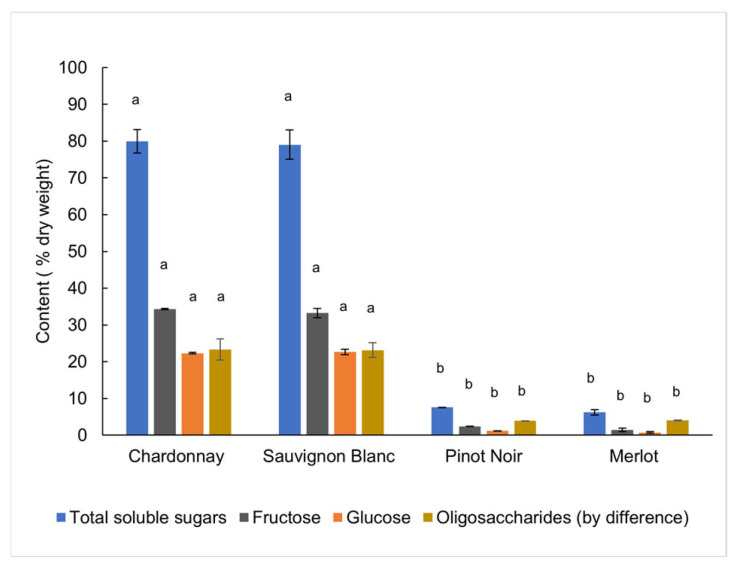
Soluble sugars determined by the Anthrone assay, glucose and fructose quantified by HPAE-PAD, and oligosaccharides estimated by difference in grape pomace from four varieties. Values are expressed as percentage of dry weight. All samples were prepared and analyzed in duplicate, and data are presented as mean ± standard deviation. Different letters (a,b) indicate statistically significant differences (*p* < 0.05) among grape varieties within the same measured component (Tukey test).

Composition of total soluble sugars, free monosaccharides, and estimated OS is illustrated in Figure 1.

Composition of soluble sugars varied markedly by grape variety and winemaking process. Pomace from white wine varieties (Chardonnay and Sauvignon Blanc) contained nearly 70% soluble carbohydrates on a dry weight basis, whereas Pinot Noir and Merlot contained substantially lower levels (7.33% and 5.9%, respectively). A similar trend was observed for free monosaccharides quantified by HPAE-PAD. Owing to the lack of commercially available analytical standards for naturally occurring OS identified in GP, the OS content was estimated by difference. This estimate represents the portion of soluble carbohydrates not attributable to free glucose/fructose and therefore likely includes OS and other small soluble carbohydrates. Using this approach, Chardonnay and Sauvignon Blanc pomaces each contained approximately 20% of non-glucose/fructose soluble carbohydrates attributable to OS (by estimation), whereas Pinot Noir and Merlot pomaces contained markedly lower levels (~3.8%), representing an approximately five-fold difference. These quantitative profiles contribute to the global effort of mapping the ‘molecular fingerprints’ of agricultural residues, aligning with the data standardization goals of the Periodic Table of Food Initiative (PTFI).

Beyond varietal differences, processing also contributes to the compositional variability observed among samples, as GP composition has been shown to vary according to grape variety, geographic origin, and winemaking procedure [21]. White GP is typically recovered after pressing and before fermentation, whereas red GP is generally obtained after fermentation and pressing. Therefore, the higher abundance of soluble carbohydrates observed in white GP is associated with the retention of carbohydrates before yeast fermentation, while red GP composition may reflect carbohydrate consumption during fermentation and prolonged contact among skins, seeds, pulp, and yeast.

Results from the literature show a similar trend for free glucose and fructose concentration in GP from Australian red and white wines [9]. For instance, red wine pomace analyzed in that study (Cabernet Sauvignon) presented lower levels of glucose and fructose than Sauvignon Blanc pomace, comparable to the ones reported here (2.1 and 2.5%, respectively, in Australian red wine pomace). While fructose values of Sauvignon Blanc pomace from Australian wine were much lower than the one reported here (19% versus 33%), the glucose value is similar between the two studies (22.7% in California Sauvignon Blanc pomace, versus 18.6% in Australian variety).

To our knowledge, no other study has attempted to estimate naturally occurring OS in different GP, especially from the Californian wine industry. The approach employed in this study to estimate OS offers preliminary values and is intended to contribute to future characterization of GP and other agrifood byproducts.

### 2.2. Identification of Oligosaccharides by LC-Q-ToF-MS

Table 1 summarizes OS identified by LC-Q-ToF-MS and their relative abundances (ion count intensity). Overall, identified OS exhibited a degree of polymerization (DP) ranging from 3 to 10 monosaccharide units and were composed of one to three monosaccharide types, including hexoses, pentoses, hexuronic acids, and N-acetyl-hexosamines.

**Figure 2:**
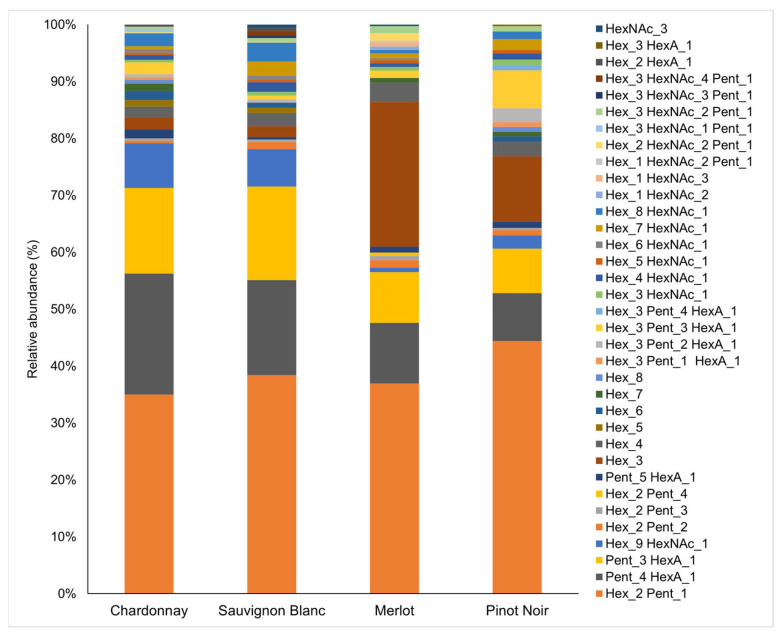
Relative abundance of oligosaccharides identified in grape pomace from four varieties, expressed as a percentage of total ion count intensity and analyzed by LC-Q-ToF-MS. Abbreviations: Hex, hexose; Pent, pentose; HexA, hexuronic acid; HexNAc, N-acetyl-hexosamine.

Across samples, 39 unique OS were identified. Chardonnay pomace exhibited the greatest diversity, with 29 identified OS, followed by Sauvignon Blanc, Merlot, and Pinot Noir pomaces, with 25, 25, and 24 compounds, respectively. Table 1 details individual OS detected in each sample, and Figure 2 highlights the relative distribution within each GP.

Hex_2_Pent_1_ was the most abundant OS identified among tested samples, comprising 45% of Pinot Noir OS and 39, 37 and 35% of Sauvignon Blanc, Merlot, and Chardonnay pomace, respectively. The second most abundant OS is Pent_4_HexA_1_, corresponding to 21% of Chardonnay pomace OS and 17, 11, and 8% of Sauvignon Blanc, Merlot, and Pinot Noir, respectively. The third most differentially abundant OS is Pent_3_HexA_1_, which accounts for 15% and 16% in Chardonnay and Sauvignon Blanc pomace, respectively, but is present at only 8% in red wine varieties. The fourth most abundant OS in white pomaces is DP 10 Hex_9_HexNAc_1_ (representing ~8% and 6.6% in Chardonnay and Sauvignon Blanc respectively). In contrast, this large OS is found only at a trace level in red pomaces (<1% in Merlot and 2.3% in Pinot Noir). In summary, four key OS cover ~80% of total OS abundance in both Chardonnay and Sauvignon Blanc: Hex_2_Pent_1_, Pent_4_HexA_1_, Pent_3_HexA_1_, and Hex_9_HexNAc1. A similar pattern was observed in red pomaces with respect to the three most abundant OS (Hex_2_Pent_1_, Pent_4_HexA_1_, Pent_3_HexA_1_). However, in contrast to white varieties, OS Hex_3_ was markedly more abundant in Merlot (25%) and Pinot Noir (12%) (versus ~2% in Chardonnay and Sauvignon Blanc) in place of Hex_9_HexNAc_1_. These dominant OS represent key structural motifs that may serve as candidates for targeted enrichment and subsequent in vitro fermentation studies linking structure to function.

The predominance of Hex_2_Pent_1_ highlights the presence of mixed hexose–pentose oligosaccharides as a major feature of the grape pomace OS profile. Such compositional diversity is important because bacterial utilization of OS is highly substrate-specific and depends on the compatibility between OS features, including monosaccharide composition, linkages, branching and degree of polymerization, and the strain-specific repertoire of carbohydrate transporters and glycoside hydrolases [22,23,24]. Nonetheless, other recent work demonstrated that OS generated from the residue remaining after beetroot sugar extraction successfully stimulated the growth of desirable commensal gut bacteria [25]. That particular set of OS provided a greater diversity of monosaccharides rather than simple repetition of one or two monomers in the chain and, therefore, was closer to the OS naturally found in GP and reported in the current study. Thus, the compositional diversity observed may influence their selective utilization by gut commensals and warrants further evaluation in biological systems.

### 2.3. Identification of Key Monosaccharides by LC-QqQ-MS

**Figure 3:**
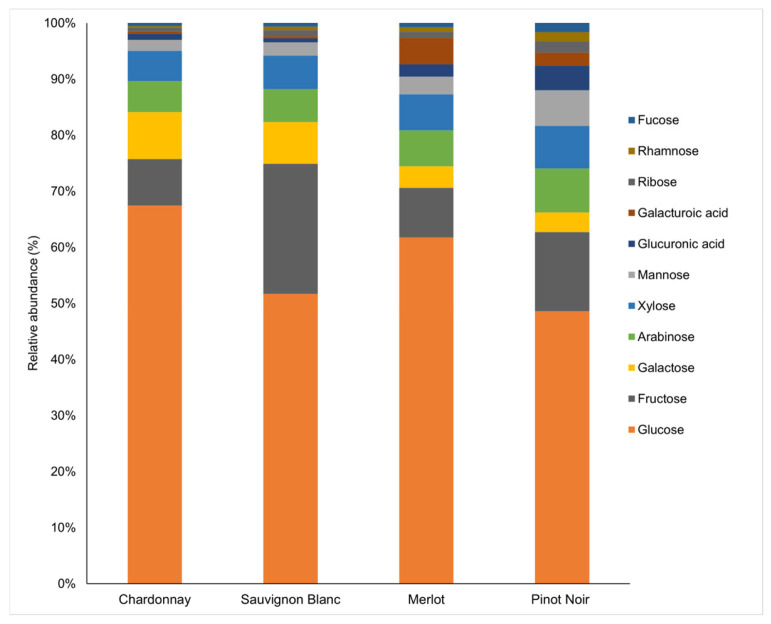
Relative abundance of monosaccharide constituting the oligosaccharide fraction in grape pomace from four varieties, expressed as a percentage of total monosaccharides analyzed. Monosaccharides were quantified by LC-QqQ-MS.

To resolve specific identities of monosaccharide building blocks, OS were subjected to acid hydrolysis followed by PMP derivatization, and released monosaccharides were analyzed by LC-QqQ-MS (Figure 3). This approach enabled the resolution and identification of structural epimers, including hexoses (galactose, glucose, and mannose); pentoses (arabinose and xylose); and 6-deoxy sugars (fucose and rhamnose). Because glucose and fructose occur in pomace extracts both as free monosaccharides and as constituents of OS, OS fractions were purified by SPE to avoid interference from free monosaccharides during OS-derived building block analysis. Previous studies using PGC-SPE have shown that, although effective at removing free monosaccharides, this approach can also lead to losses of OS, which directly undermines absolute quantification [26]. While these effects have been documented in dairy matrices, they are relevant to other complex carbohydrate-rich extracts. Accordingly, results presented here are reported as relative abundances of each monosaccharide constituent of OS rather than absolute concentrations.

Glucose was the dominant building block, accounting for ~42% in Pinot Noir (lowest) and ~67% in Chardonnay (highest). Second most abundant was fructose, with particularly high relative abundance in Sauvignon Blanc (21.1%) and Pinot Noir (12.3%), compared to Chardonnay (8.3%) and Merlot (7.4%) pomace. An elevated fructose contribution in Sauvignon Blanc suggests a higher prevalence of fructose-containing or fructan-like OS in this pomace, which may reflect varietal differences in cell wall polysaccharide composition or ripening-associated carbohydrate metabolism [27]. Among pentoses, arabinose and xylose were consistently detected, accounting for approximately 9–14% of the pool. This is consistent with the presence of arabinoxylans and arabinogalactan side chains, which are characteristic components of grape cell wall polysaccharides [28]. Galactose was present at lower but consistent levels (3–8%), supporting the contribution of galactan-rich structural motifs. Other monosaccharides, including mannose, rhamnose, and fucose, collectively contributed less than 10% of total composition but were consistently detected across all samples. The presence of rhamnose and galacturonic acid indicates contributions from pectic polysaccharides, particularly rhamnogalacturonan regions, which are abundant in grape skin and pomace cell walls [29]. Low but measurable levels of uronic acids (galacturonic and glucuronic acids) support the contribution of acidic polysaccharides, although representing a small fraction of overall OS-derived monosaccharides. These proportions are consistent with the overall relative abundance of these five monosaccharide classes in intact OS, as determined by LC-Q-ToF-MS analysis (Figure 2). Prebiotic OS have been the subject of intensive research for decades, particularly in human milk, where they have been solidified as the gold standard for prebiotics and emphasize their critical role in infant nutrition and health. Conversely, there has been minimal exploration into OS sourced from plants for human consumption, especially in agrifood byproducts. Most current prebiotics available for commercial utilization (such as fructo-, xylo-, and galacto-oligosaccharides) are somewhat simple, structurally less diverse and are often dominated by one or two monosaccharide types and do not represent the breadth of linkages and monosaccharide composition of plant biomass. Exploring alternative sources of diverse and complex OS readily available in food matrices and agrifood side products can help improve how we develop functional foods and practice sustainable agriculture. Follow-up assessment of biological activity and prebiotic potential of naturally occurring OS from GP can shed light on the complete utilization of GP as a valuable functional ingredient and improve winemaking sustainability.

### 2.4. Total Phenolic Content and Antioxidant Capacity

**Figure 4:**
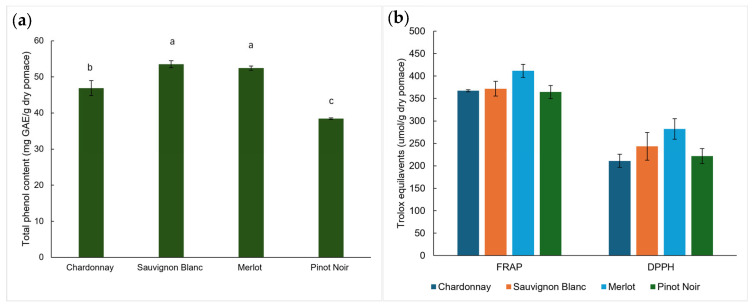
Total phenolic content (TPC) of grape pomace from four varieties (**a**), expressed as mg gallic acid equivalents (GAE) per g dry weight, and antioxidant capacity measured by DPPH and FRAP assays (**b**), expressed as µmol Trolox equivalents (TrE) per g dry weight. Values represent mean ± standard deviation (TPC, n = 3, DPPH and FRAP n = 2). Different letters in panel (**a**) indicate statistically significant differences among grape varieties (Tukey test, *p* < 0.05). No statistically significant differences were observed among grape varieties within the FRAP or DPPH assay in panel (**b**).

The total phenolic content (TPC) was measured using the Folin–Ciocalteu assay, whereas antioxidant capacity was evaluated using DPPH and FRAP assays (Figure 4).

As shown in Figure 4a, Sauvignon Blanc exhibited the highest TPC (53.51 mg GAE g^−1^ dry weight), followed closely by Merlot (52.41 mg GAE g^−1^), without a statistically significant difference. Chardonnay showed a moderate TPC (46.88 mg GAE g^−1^), whereas Pinot Noir exhibited the lowest (38.42 mg GAE g^−1^; *p* < 0.05). Antioxidant capacity assessed by DPPH and FRAP assays (Figure 4b) followed a similar trend based on mean values, but differences among varieties were not statistically significant. DPPH values ranged from 210.1 ± 14.56 µmol Trolox equivalents (TrE) g^−1^ dry weight in Chardonnay to 282.0 ± 16.72 µmol TrE g^−1^ in Merlot, while FRAP values ranged from 364.4 ± 14.5 µmol TrE g^−1^ in Pinot Noir to 411.6 ± 14.7 µmol TrE g^−1^ in Merlot. While TPC differed significantly, antioxidant capacity did not show statistically significant differences, despite Merlot exhibiting the highest mean value across both assays. These differences suggest that TPC alone does not fully explain antioxidant potential. The Folin–Ciocalteu assay provides a broad estimate of reducing compounds expressed as gallic acid equivalents and may respond to both phenolic and non-phenolic substrates [30], whereas DPPH and FRAP reflect functional radical-scavenging and ferric reducing capacities that are influenced by compound structure, stability, and bioavailability [31]. In the current study, the higher mean antioxidant capacity observed in Merlot may be partly related to its individual phenolic profile, as this variety contained the highest concentrations of several phenolic compounds associated with antioxidant capacity, including epigallocatechin-3-gallate, gallocatechin gallate, procyanidin B2, quercetin, caffeic acid, and trans-polydatin (Table 2). These results suggest that a combined assessment of TPC, functional antioxidant capacity, and targeted phenolic compounds would provide a more comprehensive interpretation of GP antioxidant potential.

Earlier investigations have indicated a greater TPC in white pomace in contrast to red pomace, primarily attributed to enhanced extraction of phenolic compounds from red grape varieties during wine fermentation [32]. Similarly, studies comparing grape skins and seeds before and after fermentative maceration, or seeds recovered after different maceration lengths, have shown that fermentation conditions can substantially modify the residual polyphenolic profile of grape-derived byproducts [33,34]. However, these studies have generally reported TPC and antioxidant capacity values lower than those observed in the current study. Importantly, Tseng and Zhao [35] demonstrated that drying methods, grape varieties, and types of byproducts (pomace and skin) greatly affect the TPC, anthocyanin, antiradical scavenging activity, and total flavonoids in Pinot Noir and Merlot pomace. This study reveals that freeze-drying retained the highest amount of TPC in all samples compared to other drying methods (oven at 40 °C, air at 25 °C and vacuum at 40 °C), which may explain the higher phenolic content in the current materials. Since samples used in the current dataset were freeze-dried, this could explain why TPC values reported here are higher overall than other values found in the literature, besides differences in processing. Another study evaluated how a different pressure and number of cycles in instant controlled pressure drop affect extractable and non-extractable polyphenols in Malbec, Syrah, and Merlot pomace [36]. It was observed that there was an increase of up to 139% in total extractable phenolics when materials were subjected to a higher pressure (0.4 MPa) and number of cycles (4). Findings derived from the present dataset, coupled with comparisons to literature values, underscore the significant influence of processing methods on TPC and antioxidant capacity. This highlights the crucial importance of processing and extraction conditions when investigating the GP composition locally and globally.

### 2.5. Low-Molecular-Weight Phenolic Compounds 

**Figure 5:**
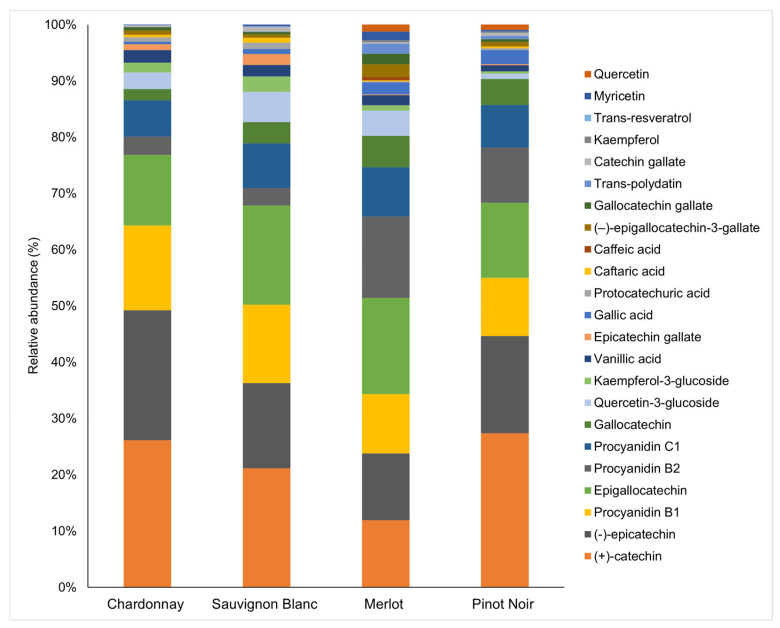
Relative abundance (%) of individual phenolic compounds quantified in grape pomace from four varieties by UPLC-DAD. Absolute concentrations for each compound are reported in Table 2.

Extracts from GP varieties were analyzed for low-molecular-weight phenolic compounds using UPLC-DAD. Table 2 reports absolute concentrations of individual phenolics, whereas Figure 5 highlights their relative distribution within each variety, providing complementary perspectives on phenolic composition. The analytical profile comprised 23 compounds spanning multiple phenolic classes, including flavan-3-ols ((+)-catechin, catechin gallate, (−)-epicatechin, epicatechin gallate, epigallocatechin, epigallocatechin-3-gallate, gallocatechin, and gallocatechin gallate), procyanidins (procyanidin B1, procyanidin B2, and procyanidin C1), flavonols (kaempferol, kaempferol-3-glucoside, quercetin, quercetin-3-glucoside, and myricetin), phenolic acids (caffeic acid, caftaric acid, gallic acid, protocatechuic acid, and vanillic acid), and stilbenes (trans-polydatin and trans-resveratrol).

**Table 2:** Quantification of phenolic compounds expressed as mg kg^−1^ on dry basis. Molecular weight (MW) is reported in g mol^−1^. All samples were analyzed in at least duplicate using an Agilent Zorbax Eclipse Plus C18 Rapid Resolution HD column (3 × 100 mm, 1.8 μm) coupled with an Agilent 1290 UPLC system. Values are reported as mean ± standard deviation, n.d = not detected. Different letters within the same row indicate statistically significant differences among grape varieties (*p* < 0.05), as determined by one-way ANOVA followed by Tukey’s multiple comparison test.

	Phenolic Compound	MW (g/mol) *	mg/kg Dry Basis			
			White Varieties		Red Varieties	
			Chardonnay	Sauvignon Blanc	Merlot	Pinot Noir
Flavan-3-ols	(+)-Catechin	290.3	1711 ± 120 ^a^	1018 ± 29 ^c^	473.2 ± 7.2 ^d^	1310 ± 67 ^b^
	Catechin gallate	442.4	19.12 ± 0.16 ^c^	39.35 ± 0.27 ^a^	10.58 ± 0.17 ^d^	25.67 ± 0.09 ^b^
	(−)-Epicatechin	290.3	1511 ± 81 ^a^	731 ± 29 ^b^	472.2 ± 5.6 ^c^	830 ± 56 ^b^
	Epicatechin gallate	442.4	67.69 ± 0.17 ^b^	92.70 ± 0.07 ^a^	5.59 ± 0.16 ^d^	9.53 ± 0.16 ^c^
	Epigallocatechin	306.3	882.3 ± 7.6 ^a^	851.5 ± 1.4 ^b^	679.16 ± 0.89 ^c^	640.4 ± 5.5 ^d^
	Epigallocatechin-3-gallate	458.4	41.85 ± 0.31 ^b^	25.71 ± 0.19 ^d^	87.71 ± 0.73 ^a^	34.17 ± 0.48 ^c^
	Gallocatechin	306.3	129.6 ± 9.2 ^c^	182.5 ± 1.3 ^b^	222.6 ± 2.4 ^a^	223.7 ± 5.8 ^a^
	Gallocatechin gallate	458.4	39.51 ± 0.21 ^b^	21.84 ± 0.16 ^c^	74.08 ± 0.51 ^a^	22.00 ± 0.31 ^c^
Procyanidin	Procyanidin B1	578.5	987.49 ± 0.71 ^a^	672.82 ± 0.25 ^b^	419.70 ± 2.8 ^d^	496.1 ± 1.5 ^c^
	Procyanidin B2	578.5	213.30 ± 0.06 ^c^	147.61 ± 0.24 ^d^	574.8 ± 4.6 ^a^	468.49 ± 0.81 ^b^
	Procyanidin C1	866.8	425.39 ± 0.53 ^a^	386.02 ± 0.22 ^b^	348.1 ± 3.0 ^d^	362.85 ± 0.11 ^c^
Flavonols	Kaempferol	286.2	n.d	n.d	13.04 ± 0.11 ^a^	8.84 ± 0.18 ^b^
	Kaempferol-3-glucoside	448.4	115.18 ± 0.20 ^b^	131.06 ± 0.19 ^a^	37.48 ± 0.20 ^c^	18.31 ± 0.14 ^d^
	Quercetin	302.2	n.d	n.d	49.41 ± 0.17 ^a^	45.65 ± 0.21 ^b^
	Quercetin-3-glucoside	464.4	192.19 ± 0.04 ^b^	259.43 ± 0.19 ^a^	177.5 ± 1.5 ^c^	44.93 ± 0.25 ^d^
	Myricetin	318.2	5.92 ± 0.09 ^c^	14.71 ± 0.07 ^b^	61.42 ± 0.37 ^a^	14.76 ± 0.10 ^b^
Phenolic acid	Caffeic acid	180.2	6.24 ± 0.01 ^b^	3.98 ± 0.09 ^c^	24.95 ± 0.06 ^a^	6.06 ± 0.12 ^b^
	Caftaric acid	312.2	28.86 ± 0.03 ^b^	40.96 ± 0.06 ^a^	7.92 ± 0.04 ^d^	12.14 ± 0.10 ^c^
	Gallic acid	170.1	29.94 ± 0.48 ^d^	43.27 ± 0.85 ^c^	85.3 ± 3.5 ^b^	117.8 ± 1.2 ^a^
	Protocatechuic acid	154.1	51.79 ± 0.27 ^b^	54.41 ± 0.11 ^a^	7.21 ± 0.17 ^d^	18.50 ± 0.03 ^c^
	Vanillic acid	168.1	146.11 ± 0.12 ^a^	99.89 ± 0.26 ^b^	71.06 ± 0.41 ^c^	53.56 ± 0.16 ^d^
Stilbenes	Trans-polydatin	390.4	1.16 ± 0.05 ^d^	3.07 ± 0.05 ^c^	71.28 ± 0.66 ^a^	28.42 ± 0.08 ^b^
	Trans-resveratrol	228.3	4.13 ± 0.10 ^b^	4.84 ± 0.04 ^a^	n.d	1.64 ± 0.07 ^c^

* Molecular weight data from [37].

Upon comparing absolute quantification data in Table 2 with relative abundance depicted in Figure 5, a clear trend emerges regarding the composition of phenolic compounds. Notably, (+)-catechin and (−)-epicatechin collectively account for up to 50% of analyzed phenolics in Chardonnay and 45% in Pinot Noir. Sauvignon Blanc pomace, on the other hand, sees (+)-catechin, (−)-epicatechin, and procyanidin B1 constituting ~50% of its profile. In Merlot, this percentage is distributed among (+)-catechin, (−)-epicatechin, procyanidin B1, and epigallocatechin.

Flavonols such as kaempferol and quercetin were exclusively detected in red varieties, whereas white varieties exhibited higher concentrations of kaempferol-3-glucoside and quercetin-3-glucoside. The glycosylation of flavonoid compounds increases their bioavailability and improves their pharmaceutical properties due to increased water solubility [38,39]. Regarding phenolic acids investigated, Chardonnay presented the highest concentration of vanillic acid (146.11 ± 0.12 mg/kg). Concentrations in Sauvignon Blanc, Merlot and Pinot Noir were 31%, 51%, and 63% lower than in Chardonnay, respectively. Pinot Noir presented the highest amount of gallic acid (117.77 ± 1.17 mg/kg). Compounds from the stilbene class were detected in small amounts in all samples, except for trans-polydatin in Merlot at 71.28 ± 0.66 mg/kg.

Phenolics are secondary plant metabolites and serve as defense mechanisms against various threats, such as herbivores, pathogens, insects, and UV radiation [40]. Some complex but hydrolysable phenolic compounds undergo biotransformation during gastrointestinal digestion into smaller, more easily absorbable molecules, enhancing their bioavailability [41]. However, only a fraction (5–10%) of ingested phenolics is absorbed in the small intestine for phase-I/II detoxification, within which some proceed to the systemic circulation with the remainder excreted back to the digestive tract after enterohepatic circulation. Along with unabsorbed phenolics, these excreted phenolic metabolites reach the colon and undergo catabolism by gut microbiota (hydrolysis, dihydroxylation, α-oxidation, hydrogenation, and decarboxylation reactions), and may then passively diffused into enterocytes or function as microbial regulators [41,42]. The findings elucidated in the present dataset reveal distinct phenolic profiles across GP, underscoring its significance as a rich repository of low-molecular-weight bioactive compounds, notably including (+)-catechin, (−)-epicatechin, procyanidin B1 and B2, and epigallocatechin. Clinical trials have associated these low-molecular-weight bioactive compounds with various health benefits, such as significant reductions in body weight, fat, and body mass index (BMI), as well as improvements in lipid profile and oxidative indices [43]. GP intake can enhance insulin sensitivity and reduce postprandial insulin levels, potentially mitigating the risk of type 2 diabetes and cardiovascular disease [44]. The Academy of Nutrition and Dietetics has published the first dietary recommendation for a flavan-3-ol intake of 400–600 mg/day to support cardiometabolic health [45]. Leveraging GP in food products could enhance their nutritional value and potentially contribute to their functional properties. Tailoring industrial application of GP from different varieties based on their phenolic profiles allows for targeted utilization in specific food products. This comprehensive dataset holds promise for customizing industrial deployment of GP sourced from diverse varieties.

## 3. Materials and Methods

### 3.1. Materials

GP from four *Vitis vinifera* L. cultivars (Chardonnay, Sauvignon Blanc, Pinot Noir, Merlot) from the 2021 vintages grown and processed in California were sourced from commercial vineyards where grapevines are purchased from nursery partners that verify plant identity at the time of purchase. Samples were provided by Sonomaceuticals, LLC (Santa Rosa, CA, USA). Samples arrived frozen and were freeze-dried in a commercial freeze dryer (VirTis model 50 SRC, SP Scientific, Warminster, PA, USA) and milled in a kitchen blender for 30 s (Vitamix 5200, Vita-Mix Corp., Cleveland, OH, USA).

HPLC-grade solvents methanol (MeOH), acetonitrile (ACN), ethanol (EtOH), and formic acid (FA); 10 N hydrochloric acid (HCl); Folin–Ciocalteu reagent; sodium carbonate anhydrous (Na_2_CO_3_); and trifluoroacetic acid (TFA) Optima™ LC/MS were purchased from Thermo Fisher Scientific (Waltham, MA, USA). Monosaccharide standards [D-(+)-glucose ≥ 99.5%, D-(+)-galactose ≥ 99%, D-(+)-mannose ≥ 99%, D-(−)-fructose ≥ 99%, L-(+)-arabinose ≥ 98%, D-(+)-xylose ≥ 95%, D-(−)-ribose ≥ 98%, L-(+)-rhamnose ≥ 99%, L-(−)-fucose ≥ 99%, D-(+)-glucuronic acid ≥ 98%, D-(+)-galacturonic acid monohydrate ≥ 95.0%], acetic acid, 1-phenyl-3-methyl-5-pyrazolone (PMP), ammonia (28.0–30.0%), polyvinylpolypyrrolidone (PVPP), sodium hydroxide (NaOH), sulfuric acid (H_2_SO_4_), 2,2′-diphenyl-1-picrylhydrazyl (DPPH), 2,4,6-Tris(2-pyridyl)-s-triazine (TPTZ), ferric chloride (FeCl_3_), Trolox, sodium acetate (C_2_H_3_NaO_2_), and chloroform were purchased from Sigma-Aldrich (St. Louis, MO, USA). Anthrone reagent was purchased from Alfa Aesar (Haverhill, MA, USA). The United States Pharmacopeia (USP) reference standard of purified grape seed oligomeric proanthocyanidins, analytical standards (purity ≥ 98%) of gallic acid, (+)-catechin, (−)-epicatechin, (−)-gallocatechin, protocatechuic acid, caftaric acid, procyanidin B1, (−)-epigallocatechin, vanillic acid, caffeic acid, procyanidin B2, (−)-epigallocatechin gallate, procyanidin C1, (−)-gallocatechin gallate, trans-polydatin, (−)-epicatechin gallate, (−)-catechin gallate, quercetin-3-glucoside, trans-resveratrol, kaempferol-3-glucoside, myricetin, quercetin, and kaempferol were obtained from Millipore Sigma (Burlington, MA, USA). Nanopure water was generated from a Milli-Q system (Millipore, Bedford, MA, USA).

### 3.2. Characterization of Carbohydrates

#### 3.2.1. Extraction of Soluble Sugars

Soluble sugars (monosaccharides and OS) were extracted by combining 15 mg of each GP sample with 500 µL of nanopure water in an Eppendorf tube, shaken in a thermomixer (ThermoMixer C, Eppendorf, Hamburg, Germany) at 100 °C, 1000 rpm for 10 min, centrifuged at 15,000× *g* for 10 min. Supernatant was separated, and the pellet was extracted two more times, combining supernatants. Extracts were filtered (Agilent Captiva Econo Filter, PES, 13 mm, 0.2 μm) (Agilent Technologies, Santa Clara, CA, USA) and dried in a centrifugal evaporator (Genevac miVac concentrator, SP Scientific, Warminster, PA, USA).

#### 3.2.2. Quantification of Total Carbohydrates

The total carbohydrate content was assessed by the Anthrone method with modifications [46]. In a 96-well microplate, 40 μL of extracts produced in Section 3.2.1 was combined with 100 μL of Anthrone reagent (2 mg/mL (*w*/*v*) in cold 98% sulfuric acid), incubated for 3 min at 92 °C in a Thermo Scientific Precision 280 Series water bath (Thermo Fisher Scientific, Waltham, MA, USA), and then cooled for 5 min at room temperature before measuring the absorbance with a SpectraMax M5 UV/Vis spectrophotometer (Molecular Devices, San Jose, CA, USA) at 630 nm. Total carbohydrate quantification calculations were based on a glucose standard curve ranging from 25 to 300 mg/L.

#### 3.2.3. Quantification of Free Monosaccharides by HPAE-PAD

Free glucose and fructose were quantified by high-performance anion-exchange chromatography with pulsed amperometric detection (HPAE-PAD) on a Thermo Dionex ICS-5000 system, based on a method with modifications [47]. Extracts produced in Section 3.2.1 were injected into a DionexCarboPac PA200 guard column (3 × 50 mm) and a Dionex CarboPac PA200 analytical column (3 × 250 mm) (Thermo Fisher Scientific, Waltham, MA, USA), and chromatographic separation was carried out with a 12 min gradient elution (from 0.6 to 25% B in 12 min), with a 0.5 mL min^−1^ flow rate. Solvent consisted of A: 100% water and B: 200 mM sodium hydroxide (NaOH). Calibration curves were prepared using glucose and fructose standards ranging from 0.0001 to 0.006 mg/mL.

#### 3.2.4. Estimation of Oligosaccharides

Due to a lack of analytical standards for absolute quantification of newly discovered OS, an approach was used to estimate by difference. This consisted of quantifying total extracted soluble sugars (total soluble sugars) and subtracting the total amount of free glucose and free fructose, as shown in Equation (1). The aim is to combine it with compositional elucidation provided by LC-MS to gain a comprehensive understanding of OS differences within GP varieties.

(1) $$\mathrm{Estimated}\;\mathrm{oligosaccharides}=\mathrm{total}\;\mathrm{soluble}\;\mathrm{sugars}-\left(\mathrm{free}\;\mathrm{glucose}+\mathrm{free}\;\mathrm{fructose}\right)$$

#### 3.2.5. Oligosaccharide Isolation Prior to LC-MS Analysis

GP extracts (Section 3.2.1) were purified through a multi-step solid-phase extraction (SPE). Adsorbent PVPP was used to bind phenolic compounds, as described [48,49]. PVPP was added to GP extracts at a concentration of 5 mg PVPP/mL and shaken for 15 min in a Thermomixer (ThermoMixer C, Eppendorf, Hamburg, Germany). The mixture was centrifuged at 4000× *g* for 5 min, and supernatant was separated. Additional PVPP was added to the supernatant (5 mg PVPP/mL), and the process was repeated. The supernatant was dried in a centrifugal evaporator.

Next, extracts were purified using SPE reversed-phase octadecyl (C18) and porous graphitized carbon (PGC). C18 cartridges (DISCOVERY DSC-18 SPE, 1000 mg; Sigma-Aldrich) were conditioned with ACN and nanopure water. Then, 500 µL of PVPP purified extracts was loaded onto C18 cartridges and washed with nanopure water. The flow-through was collected and loaded on a conditioned PGC cartridge (Supelclean™ Envi™ Carb, 500 mg; Sigma-Aldrich). PGC cartridges were washed with nanopure water, and OS were eluted using 40% ACN, and 0.1% FA in water. Purified extracts were dried in a centrifugal evaporator.

#### 3.2.6. Oligosaccharide Analysis Using LC-Q-ToF-MS

Individual OS compositions were obtained with an Agilent 6520 NanoChip LC-Q-ToF-MS (Santa Clara, CA, USA). OS separation was achieved with a microfluidic high-performance liquid chromatography (HPLC) PGC chip containing enrichment (4 mm, 40 nL) and analytical (75 μm × 43 mm) column, nanoelectrospray tip, and binary solvent gradient of solvent A (3% ACN, 0.1% TFA in water) and solvent B (90% ACN, 0.1% TFA in water) based on a previously optimized method [50]. The gradient was 0–16% B at 2.5–20 min, 16–44% B at 20–30 min, 44–100% B at 30–35 min, 100% B at 35–45 min, and 100–0% B from 45 to 45.1 min, followed by 10 min re-equilibration of 100% A.

A mass spectrometer (MS) was operated in the positive ionization mode with a range of 450–2500 Da and an electrospray capillary voltage of 1800–1900 V. Reference masses of 922.009 and 1221.991 Da (ESI-ToF Tuning Mix G1969-85000, Agilent Technologies, Santa Clara, CA, USA) provided continuous internal calibration. We performed tandem MS/MS with tandem fragmented peaks selected by automated precursor selection setting with a threshold of 200 ion counts for MS and 5 ion counts for MS/MS. The ramped collision energy slope was 1.3 with an offset of −3.6 V, medium isolation width for MS/MS, and acquisition rate of 1 spectra/s. Each spectrum was manually examined. Molecular masses were confirmed with Agilent MassHunter Qualitative Analysis B.07.00 software using molecular feature extraction and a maximum tolerance of 20 ppm.

#### 3.2.7. Analysis of Oligosaccharide Building Blocks by LC-QqQ-MS

Isolated OS (Section 3.2.5) were subjected to acid hydrolysis to release their constituent monosaccharides. Aliquots of 50 µL of each OS sample were hydrolyzed in aqueous 4M TFA solution in a boiling water bath for 40 min (Microprocessor Controlled 280 Series, Precision Inc., Winchester, VA, USA) [51]. Following hydrolysis, samples were diluted 100 times, thoroughly dried using a centrifugal evaporator, and reconstituted in water prior to PMP derivatization and LC-QqQ-MS analysis.

Calibration curves (glucose, galactose, mannose, fructose, arabinose, xylose, ribose, rhamnose, fucose, glucuronic acid, galacturonic acid) were prepared at concentrations of 0.0001–0.5 mg/mL. Both standards and hydrolyzed samples were PMP-derivatized, modified from [52] as follows. Briefly, 50 µL of standards or hydrolyzed samples was mixed with 200 µL ammonia solution in water (28.0–30.0%), and we added 200 µL 0.5 M PMP in MeOH, incubated at 70 °C (in water bath) for 30 min and dried using vacuum centrifugation. Dried samples were reconstituted in 200 µL water and washed 3 times with 500 µL chloroform to remove excess PMP. Next, 40 µL from each standard or sample was mixed with 160 µL nanopure water, and 1 µL was used for QqQ-MS analysis. An Agilent 6470 QqQ MS equipped with an Agilent 1260 infinity HPLC system and an Agilent ZORBAX Eclipse Plus C18 column (2.1 mm × 30 mm i.d., 1.8 μm particle size) (Agilent Technologies, Santa Clara, CA, USA) was used. Mobile phase A was 25 mM ammonium acetate in 5% ACN in water (*v*/*v*) with pH adjusted to 8.2–8.5 using 25% ammonia solution. Mobile phase B was 95% ACN in water (*v*/*v*). Separation of PMP-labeled monosaccharides was performed using a 40 min binary gradient of 0–5 min, 0–0% B; 5–10 min, 0–5% B; 10–25.0 min, 5–8% B; 25–30 min, 8–14% B; 30–33, 14–18% B; 33–38, 18–100%; 38–40 min, 100–0% B, with an extra 3 min post-gradient equilibration time, flow rate of 0.5 mL/min, and column temperature of 35 °C. An MS system was equipped with an orthogonal ESI, operated in the positive ion mode and with multiple reaction monitoring (MRM). MS parameters were as follows: the drying gas and sheath gas temperature were 290 °C and 300 °C, respectively. The drying gas and sheath gas flow rate were 11 L/min and 12 L/min, respectively. At nebulizer pressure 30 psi, the capillary voltage was 1800 V. Amplitudes of RF voltage for high-pressure and low-pressure ion funnels were 150 V and 60 V, respectively. For scanning product ions, the mass range was set at *m*/*z* 50–600 using the Agilent MassHunter qualitative 10.0 (Agilent Technologies, Santa Clara, CA, USA).

### 3.3. Characterization of Phenolics

#### 3.3.1. Extraction of Phenolic Compounds

Ethanolic extracts were produced by combining 10 mg of each GP with 5 mL of 50% EtOH-water, sonicated for 20 min Turbo Sonic 6000, Lyman Products Corporation, Middletown, CT, USA) and centrifuged at 4000 rpm for 10 min (Sorvall Legend X1 Centrifuge, Thermo Fisher Scientific, Waltham, MA, USA). Supernatants were used for determination of the total phenol content (Section 3.3.2) and ferric reducing antioxidant power (FRAP) assay (Section 3.3.3). Methanolic extracts were produced by combining 200 mg of each GP with 10 mL of 50% MeOH water, sonicated for 20 min and centrifuged at 4000 rpm for 10 min. Supernatants were used for 2,2′-Diphenyl-1-picrylhydrazyl radical (DPPH) (Section 3.3.4) and quantification of selected low-molecular-weight phenolic compounds by UPLC-DAD (Section 3.3.5).

#### 3.3.2. Quantification of Total Phenol Content

The total phenol content (TPC) was measured as described by [53] with modifications. Ethanolic extracts were diluted by combining 500 µL in a 10 mL volumetric flask with 2.5 mL of Folin–Ciocalteu reagent (diluted 1:10 with water) and 2 mL of sodium carbonate (75 g/L) and filling up to 10 mL with distilled water. Samples were incubated in the dark for 2 h before absorbance was measured at 760 nm on a Genesys 10S UV-Vis Spectrophotometer (Thermo Fisher Scientific, Waltham, MA, USA). A calibration curve was prepared using gallic acid (1.25–125 mg/L).

#### 3.3.3. Ferric Reducing Antioxidant Power (FRAP)

Phenolic compounds were oxidized by Fe (III) in the FRAP assay. The determination protocol was adopted from [54]. Fifty microliters of ethanolic extracts were combined with 950 µL of FRAP assay (10 mM TPTZ dissolved in 40 mM HCl:20 mM FeCl_3_:300 mM acetate buffer at pH 3.6, 1:1:10 by vol.), incubated at 37 °C for 30 min (Precision SWB 15 shaking water bath, Thermo Fisher Scientific, Waltham, MA, USA). Absorbance was measured at 595 nm (BioTek Synergy H1 microplate reader, Agilent Technologies, Santa Clara, CA, USA). EtOH and Trolox were used in the blank and standard curves (0.1 to 1.0 µmol/mL), respectively. Results were expressed as μmol TrE/g GP.

#### 3.3.4. 2,2′-Diphenyl-1-Picrylhydrazyl Radical (DPPH)

Antioxidant capacity in the DPPH assay was measured based on [54]. Briefly, 5 µL of methanolic extract was added to 195 µL methanolic DPPH solution (40 mg/L) and incubated in the dark for 30 min. Phenolics reduce the DPPH radical as a loss of absorbance at 515 nm, measured on a BioTek Synergy H1 microplate reader (Agilent Technologies, Santa Clara, CA, USA). Trolox was used in standard solutions in the 0.1 to 1.0 µmol/mL concentration range. Radical scavenging activity was expressed as µmol Trolox equivalent (TrE)/g GP on the percentage inhibition.

#### 3.3.5. Quantification of Selected Low-Molecular-Weight Phenolic Compounds by UPLC-DAD

Selected phenolic quantification targeting low-molecular-weight compounds was analyzed following protocols provided in [55] with modifications listed in [50]. One milliliter of methanolic extract was filtered through a 0.45 µm membrane (Thermo Fisher Scientific, Waltham, MA, USA) prior to UPLC injection. Separation and quantification of phenolic compounds were achieved on an Agilent Zorbax Eclipse Plus C18 Rapid Resolution HD (3 × 100 mm, 1.8 µm) installed on an Agilent 1290 UPLC coupled with a DAD (Agilent Technologies, Santa Clara, CA, USA). The mobile phase consisted of solvent A (0.2% FA in nanopure water) and solvent B (50% ACN, 50% MeOH) and the gradient was from 95% A to 65% A in 30 min; 65% A to 60% A in 10 min; and 60% A to 95% A in 2 min. The injection volume was 5 µL and flow rate 0.5 mL/min. Individual compounds were quantified at 280 nm (phenolic acids and flavanols), 320 nm (flavonols), and 349 nm (stilbenes) using respective external standard calibration curves.

### 3.4. Statistical Analysis

Each GP sample was independently extracted in duplicate or triplicate from the same pomace lot and each extract was analyzed in duplicate or triplicate, depending on the assay. Total carbohydrate quantification, glucose, fructose, antioxidant capacity (DPPH and FRAP), OS and phenolic profiles were measured using duplicate biological and technical replicates, while the total phenolic content was extracted and analyzed in triplicate. All data are expressed as mean ± standard deviation (SD). One-way analysis of variance (ANOVA) followed by Tukey’s multiple comparisons test was performed in R version 4.5.1 to evaluate differences among varieties, with significance set at *p* < 0.05.

## 4. Conclusions

This study provides a comprehensive molecular characterization of GP derived from four economically important Californian varieties: Chardonnay, Sauvignon Blanc, Pinot Noir, and Merlot. By applying high-resolution analytical platforms, this research reveals a level of molecular complexity that extends beyond traditionally recognized fiber and antioxidants, establishing GP as a significant underutilized source of structurally diverse metabolites.

Across all samples, the distribution of 39 unique OS spanning multiple DP was resolved, demonstrating that the molecular signature of the material is a function of the specific variety and its corresponding processing. White wine pomaces, particularly Chardonnay, exhibited greater OS structural diversity, while red pomaces displayed distinct carbohydrate profiles. Analysis of OS building blocks indicated the presence of neutral, deoxy, and acidic monosaccharides associated with plant cell-wall polysaccharides.

In parallel, the quantitative profiling of 23 low-molecular-weight phenolics, including flavan-3-ols such as (+)-catechin and (−)-epicatechin, confirms that these side streams are rich reservoirs of bioactive metabolites.

The co-occurrence of structurally complex OS and phenolic compounds within the same matrix positions GP as a multifunctional ingredient, capable of providing the chemical diversity required for high-value upcycling. Overall, these findings advance the understanding of GP as more than an industrial byproduct, providing a compositional framework that supports its targeted valorization. By resolving the specific distributions of phenolics and OS, this work contributes to providing a solid chemical foundation required to transition from bulk residue disposal to the targeted recovery of high-value molecular ingredients.

Such an approach contributes to improved sustainability in winemaking through the high-value utilization of grape-derived residues and aligns with broader efforts to promote circular and resource-efficient food systems. These data provide a compositional reference for future studies evaluating the nutritional and functional properties of GP-derived ingredients.

## Figures and Tables

**Table 1:** Oligosaccharides identified in white and red grape pomace by LC-Q-ToF-MS. The table reports the composition and oligosaccharide features, such as mass-to-charge ratio (*m*/*z*), degree of polymerization (DP), and relative abundance (ion count intensity). Compositions are denoted by the number of each monosaccharide type present: Hex, hexose; Pent, pentose; HexA, hexuronic acid; HexNAc, N-acetyl-hexosamine. Ion count intensity was determined by manual peak integration. n.d = non detected. The total number of unique compositions identified for each grape pomace variety is reported at the bottom of the table.

Identified Compound	*m*/*z*	DP	Ion Count Intensity			
			White Varieties		Red Varieties	
			Chardonnay	Sauvignon Blanc	Merlot	Pinot Noir
Hex_2_Pent_1_	475.1658	3	1.05 × 10^6^	8.47 × 10^5^	4.53 × 10^5^	7.40 × 10^5^
Hex_2_Pent_2_	607.2081	4	1.34 × 10^4^	2.77 × 10^4^	1.63 × 10^4^	1.55 × 10^4^
Hex_2_Pent_3_	739.2504	5	1.14 × 10^4^	9.51 × 10^3^	8.99 × 10^3^	6.20 × 10^3^
Hex_2_Pent_4_	871.2927	6	n.d	n.d	7.15 × 10^3^	n.d
Hex_3_Pent_2_	769.2609	5	1.59 × 10^4^	n.d	n.d	n.d
Pent_3_HexA_1_	573.1663	4	4.51 × 10^5^	3.62 × 10^5^	1.09 × 10^5^	1.30 × 10^5^
Pent_4_HexA_1_	705.2086	5	6.37 × 10^5^	3.68 × 10^5^	1.31 × 10^5^	1.40 × 10^5^
Pent_5_HexA_1_	855.2615	6	4.84 × 10^4^	1.03 × 10^4^	1.29 × 10^4^	1.85 × 10^4^
Hex_3_	505.1763	3	6.25 × 10^4^	4.18 × 10^4^	3.12 × 10^5^	1.92 × 10^5^
Hex_4_	667.2291	4	5.64 × 10^4^	5.18 × 10^4^	4.17 × 10^4^	4.18 × 10^4^
Hex_5_	829.2819	5	3.50 × 10^4^	2.01 × 10^4^	n.d	n.d
Hex_6_	991.3347	6	4.91 × 10^4^	1.92 × 10^4^	n.d	1.59 × 10^4^
Hex_7_	1153.388	7	3.68 × 10^4^	n.d	1.02 × 10^4^	1.36 × 10^4^
Hex_8_	1315.44	8	2.07 × 10^4^	n.d	n.d	1.37 × 10^4^
Hex_2_Pent_3_HexA_1_	915.2825	6	n.d	1.29 × 10^4^	n.d	n.d
Hex_1_Pent_3_HexA_1_	735.2191	5	1.38 × 10^4^	n.d	n.d	n.d
Hex_3_Pent_1_HexA_1_	813.2507	5	1.59 × 10^4^	n.d	n.d	1.43 × 10^4^
Hex_3_Pent_2_HexA_1_	945.293	6	1.34 × 10^4^	1.22 × 10^4^	n.d	4.09 × 10^4^
Hex_3_Pent_3_HexA_1_	1077.335	7	6.19 × 10^4^	1.58 × 10^4^	1.54 × 10^4^	1.11 × 10^5^
Hex_3_Pent_4_HexA_1_	1209.378	8	n.d	n.d	n.d	1.59 × 10^4^
Hex_3_HexNAc_1_	708.2557	4	1.43 × 10^4^	1.36 × 10^4^	8.23 × 10^3^	1.52 × 10^4^
Hex_4_HexNAc_1_	870.3085	5	2.55 × 10^4^	3.76 × 10^4^	7.63 × 10^3^	1.83 × 10^4^
Hex_5_HexNAc_1_	1032.361	6	1.12 × 10^4^	9.52 × 10^3^	6.75 × 10^3^	1.02 × 10^4^
Hex_6_HexNAc_1_	1194.414	7	1.97 × 10^4^	1.52 × 10^4^	4.35 × 10^3^	n.d
Hex_7_HexNAc_1_	1356.467	8	1.48 × 10^4^	5.49 × 10^4^	1.04 × 10^4^	3.16 × 10^4^
Hex_8_HexNAc_1_	1518.52	9	6.83 × 10^4^	7.36 × 10^4^	7.46 × 10^3^	2.23 × 10^4^
Hex_9_HexNAc_1_	1680.573	10	2.37 × 10^5^	1.45 × 10^5^	9.95 × 10^3^	3.91 × 10^4^
Hex_1_HexNAc_2_	587.2295	3	n.d	n.d	7.09 × 10^3^	n.d
Hex_1_HexNAc_3_	772.2983	4	n.d	n.d	9.21 × 10^3^	n.d
Hex_1_HexNAc_2_Pent_1_	701.2612	4	n.d	n.d	4.18 × 10^3^	n.d
Hex_2_HexNAc_2_Pent_1_	881.3246	5	7.20 × 10^3^	4.27 × 10^3^	1.54 × 10^4^	n.d
Hex_3_HexNAc_1_Pent_1_	840.298	5	1.65 × 10^4^	n.d	n.d	n.d
Hex_3_HexNAc_2_Pent_1_	1043.377	6	1.08 × 10^4^	1.36 × 10^4^	1.53 × 10^4^	1.60 × 10^4^
Hex_3_HexNAc_3_Pent_1_	1246.4568	7	n.d	1.06 × 10^4^	n.d	n.d
Hex_3_HexNAc_4_Pent_1_	1449.536	8	n.d	1.80 × 10^4^	n.d	n.d
Hex_2_HexA_1_	519.1556	3	1.17 × 10^4^	1.09 × 10^4^	n.d	n.d
Hex_3_HexA_1_	681.2084	4	n.d	n.d	n.d	4.43 × 10^3^
HexNAc_3_	610.2455	3	n.d	n.d	3.60 × 10^3^	n.d
HexNAc_1_Pent_2_HexA_1_	644.2034	4	7.88 × 10^3^	n.d	n.d	n.d
Total number of unique compositions			29	25	25	24

## Data Availability

The original contributions presented in this study are included in the article. Further inquiries can be directed to the corresponding author.

## Abbreviations

The following abbreviations are used in this manuscript:

ACN	Acetonitrile
ANOVA	Analysis of variance
BMI	Body Mass Index
C18	Octadecyl solid-phase extraction cartridge
DP	Degree of polymerization
DPPH	2,2′-Diphenyl-1-picrylhydrazyl
ESI	Electrospray ionization
EtOH	Ethanol
FA	Formic acid
FeCl_3_	Ferric chloride
FRAP	Ferric reducing antioxidant power
GAE	Gallic acid equivalents
GP	Grapepomace
HCl	Hydrochloric acid
Hex	Hexose
HexA	Hexuronic acid
HexNAc	N-acetyl-hexosamine
HPAE-PAD	High-performance anion-exchange chromatography with pulsed amperometric detection
HPLC	High-performance liquid chromatography
LC-MS	Liquid chromatography mass spectrometry
LC-QqQ-MS	Liquid chromatography triple quadrupole mass spectrometry
LC-Q-ToF-MS	Liquid chromatography quadrupole time-of-flight mass spectrometry
MeOH	Methanol
MS	Mass spectrometry
MS/MS	Tandem mass spectrometry
MW	Molecular weight
Na_2_CO_3_	Sodium carbonate
NaOH	Sodium hydroxide
OS	Oligosaccharides
PGC	Porous graphitized carbon
PMP	1-Phenyl-3-methyl-5-pyrazolone
PTFI	Periodic Table of Food Initiative
PVPP	Polyvinylpolypyrrolidone
SPE	Solid-phase extraction
TFA	Trifluoroacetic acid
TPC	Total phenolic content
TPTZ	2,4,6-Tris(2-pyridyl)-s-triazine
TrE	Trolox equivalents
UPLC-DAD	Ultra-performance liquid chromatography with diode array detection
USP	United States Pharmacopeia
UV	Ultraviolet
× *g*	Relative centrifugal force

## References

[1] USDA, NASS (2025). 2024 California Grape Crush Final Report.

[2] Boussetta N., Lanoisellé J.-L., Bedel-Cloutour C., Vorobiev E. (2009). Extraction of Soluble Matter from Grape Pomace by High Voltage Electrical Discharges for Polyphenol Recovery: Effect of Sulphur Dioxide and Thermal Treatments. J. Food Eng..

[3] Moreno A.D., Ballesteros M., Negro M.J. (2020). Biorefineries for the Valorization of Food Processing Waste. The Interaction of Food Industry and Environment.

[4] Hoxha L., Taherzadeh M., Marangon M. (2025). Sustainable Repurposing of Grape Marc: Potential for Bio-Based Innovations. Waste Manag..

[5] Natolino A., Manfé L., Voce S., Barp L. (2025). Polysaccharides Recovery by Subcritical Water as an Additional Step for a Biorefinery Valorisation of Grape Marc with Pressurized Fluids. LWT.

[6] Martinez G.A., Puccio S., Domingos J.M.B., Morselli E., Gioia C., Marchese P., Galletti A.M.R., Celli A., Fava F., Bertin L. (2022). Upgrading Grape Pomace Contained Ethanol into Hexanoic Acid, Fuel Additives and a Sticky Polyhydroxyalkanoate: An Effective Alternative to Ethanol Distillation. Green Chem..

[7] Ibn Ferjani A., Jeguirim M., Jellali S., Limousy L., Courson C., Akrout H., Thevenin N., Ruidavets L., Muller A., Bennici S. (2019). The Use of Exhausted Grape Marc to Produce Biofuels and Biofertilizers: Effect of Pyrolysis Temperatures on Biochars Properties. Renew. Sustain. Energy Rev..

[8] Hixson J.L., Jacobs J.L., Wilkes E.N., Smith P.A. (2016). Survey of the Variation in Grape Marc Condensed Tannin Composition and Concentration and Analysis of Key Compositional Factors. J. Agric. Food Chem..

[9] Corbin K.R., Hsieh Y.S.Y., Betts N.S., Byrt C.S., Henderson M., Stork J., DeBolt S., Fincher G.B., Burton R.A. (2015). Grape Marc as a Source of Carbohydrates for Bioethanol: Chemical Composition, Pre-Treatment and Saccharification. Bioresour. Technol..

[10] Deng Q., Penner M.H., Zhao Y. (2011). Chemical Composition of Dietary Fiber and Polyphenols of Five Different Varieties of Wine Grape Pomace Skins. Food Res. Int..

[11] Yu J., Ahmedna M. (2013). Functional Components of Grape Pomace: Their Composition, Biological Properties and Potential Applications. Int. J. Food Sci. Tech..

[12] Han Y., Yang C., Tian X., Shi X., Wang H., Li H. (2025). Grape Pomace Polyphenol Extract Alleviates Obesity in Mice and Improves Gut Microbiota and Short Chain Fatty Acids. Foods.

[13] Castillo A., Celeiro M., Rubio L., Bañobre A., Otero-Otero M., Garcia-Jares C., Lores M. (2022). Optimization of Bioactives Extraction from Grape Marc via a Medium Scale Ambient Temperature System and Stability Study. Front. Nutr..

[14] Cejudo-Bastante C., Arjona-Mudarra P., Fernández-Ponce M.T., Casas L., Mantell C., Martínez de la Ossa E.J., Pereyra C. (2021). Application of a Natural Antioxidant from Grape Pomace Extract in the Development of Bioactive Jute Fibers for Food Packaging. Antioxidants.

[15] Gibson G.R., Hutkins R., Sanders M.E., Prescott S.L., Reimer R.A., Salminen S.J., Scott K., Stanton C., Swanson K.S., Cani P.D. (2017). Expert Consensus Document: The International Scientific Association for Probiotics and Prebiotics (ISAPP) Consensus Statement on the Definition and Scope of Prebiotics. Nat. Rev. Gastroenterol. Hepatol..

[16] Zhang A., Wu Q., Mayne J., Ning Z., Qin H., Dewar A., Figeys D. (2025). Microbiome-Dependent Functional Responses to Structurally Distinct Oligosaccharides Revealed by Metaproteomics. npj Biofilms Microbiomes.

[17] Cosme P., Rodríguez A.B., Espino J., Garrido M. (2020). Plant Phenolics: Bioavailability as a Key Determinant of Their Potential Health-Promoting Applications. Antioxidants.

[18] Soto-Hernández M., García-Mateos R., Palma-Tenango M. (2019). Plant Physiological Aspects of Phenolic Compounds.

[19] Jarvis A., Gallo-Franco J., Portilla J., German B., Debouck D., Rajasekharan M., Khoury C., Herforth A., Ahmed S., Tohme J. (2024). Periodic Table of Food Initiative for Generating Biomolecular Knowledge of Edible Biodiversity. Nat. Food.

[20] Lange M.C., Li R., Apolzan J.W., Huber P.R., Steliotes E., Robertson K., Wilson N.L.W., Jain K., Ramnath R., Roe B.E. (2025). Ontologies Relevant for Improving Data Interoperability for Food Loss and Waste: A Review and Research Agenda. Clean. Responsible Consum..

[21] Jin Q., O’Hair J., Stewart A.C., O’Keefe S.F., Neilson A.P., Kim Y.-T., McGuire M., Lee A., Wilder G., Huang H. (2019). Compositional Characterization of Different Industrial White and Red Grape Pomaces in Virginia and the Potential Valorization of the Major Components. Foods.

[22] Koropatkin N.M., Cameron E.A., Martens E.C. (2012). How Glycan Metabolism Shapes the Human Gut Microbiota. Nat. Rev. Microbiol..

[23] Rivière A., Moens F., Selak M., Maes D., Weckx S., De Vuyst L. (2014). The Ability of Bifidobacteria To Degrade Arabinoxylan Oligosaccharide Constituents and Derived Oligosaccharides Is Strain Dependent. Appl. Environ. Microbiol..

[24] Kelly S.M., Munoz-Munoz J., van Sinderen D. (2021). Plant Glycan Metabolism by Bifidobacteria. Front. Microbiol..

[25] Paviani B., Masarweh C., Bhattacharya M., Ozturk G., Castillo J., Couture G., Lebrilla C.B., Mills D.A., Barile D. (2024). Eat Your Beets: Conversion of Polysaccharides into Oligosaccharides for Enhanced Bioactivity. Int. J. Biol. Macromol..

[26] Robinson R.C., Colet E., Tian T., Poulsen N.A., Barile D. (2018). An Improved Method for the Purification of Milk Oligosaccharides by Graphitised Carbon-Solid Phase Extraction. Int. Dairy. J..

[27] Blanch M., Sanchez-Ballesta M.T., Escribano M.I., Merodio C. (2011). Fructo-Oligosaccharides in Table Grapes and Response to Storage. Food Chem..

[28] Williams D.L., Schückel J., Vivier M.A., Buffetto F., Zietsman A.J.J. (2019). Grape Pomace Fermentation and Cell Wall Degradation by Kluyveromyces Marxianus Y885. Biochem. Eng. J..

[29] Jones-Moore H., Jelley R., Marangon M., Fedrizzi B. (2021). The Polysaccharides of Winemaking: From Grape to Wine. Trends Food Sci. Technol..

[30] Everette J.D., Bryant Q.M., Green A.M., Abbey Y.A., Wangila G.W., Walker R.B. (2010). Thorough Study of Reactivity of Various Compound Classes toward the Folin−Ciocalteu Reagent. J. Agric. Food Chem..

[31] Lang Y., Gao N., Zang Z., Meng X., Lin Y., Yang S., Yang Y., Jin Z., Li B. (2024). Classification and Antioxidant Assays of Polyphenols: A Review. J. Future Foods.

[32] de la Cerda-Carrasco A., López-Solís R., Nuñez-Kalasic H., Peña-Neira Á., Obreque-Slier E. (2015). Phenolic Composition and Antioxidant Capacity of Pomaces from Four Grape Varieties (*Vitis vinifera* L.). J. Sci. Food Agric..

[33] Bosso A., Cassino C., Motta S., Panero L., Tsolakis C., Guaita M. (2020). Polyphenolic Composition and In Vitro Antioxidant Activity of Red Grape Seeds as Byproducts of Short and Medium-Long Fermentative Macerations. Foods.

[34] Guaita M., Bosso A. (2019). Polyphenolic Characterization of Grape Skins and Seeds of Four Italian Red Cultivars at Harvest and after Fermentative Maceration. Foods.

[35] Tseng A., Zhao Y. (2012). Effect of Different Drying Methods and Storage Time on the Retention of Bioactive Compounds and Antibacterial Activity of Wine Grape Pomace (Pinot Noir and Merlot). J. Food Sci..

[36] Martínez-Meza Y., Pérez-Jiménez J., Castaño-Tostado E., Pérez-Ramírez I.F., Alonzo-Macías M., Reynoso-Camacho R. (2022). Instant Controlled Pressure Drop as a Strategy To Modify Extractable and Non-Extractable Phenolic Compounds: A Study in Different Grape Pomace Materials. J. Agric. Food Chem..

[37] Neveu V., Perez-Jiménez J., Vos F., Crespy V., du Chaffaut L., Mennen L., Knox C., Eisner R., Cruz J., Wishart D. (2010). Phenol-Explorer: An Online Comprehensive Database on Polyphenol Contents in Foods. Database.

[38] Slámová K., Kapešová J., Valentová K. (2018). “Sweet Flavonoids”: Glycosidase-Catalyzed Modifications. Int. J. Mol. Sci..

[39] Jan R., Khan M., Asaf S., Lubna, Asif S., Kim K.-M. (2022). Bioactivity and Therapeutic Potential of Kaempferol and Quercetin: New Insights for Plant and Human Health. Plants.

[40] Metsämuuronen S., Sirén H. (2019). Bioactive Phenolic Compounds, Metabolism and Properties: A Review on Valuable Chemical Compounds in Scots Pine and Norway Spruce. Phytochem. Rev..

[41] Ferreira-Santos P., Nobre C., Rodrigues R.M., Genisheva Z., Botelho C., Teixeira J.A. (2024). Extraction of Phenolic Compounds from Grape Pomace Using Ohmic Heating: Chemical Composition, Bioactivity and Bioaccessibility. Food Chem..

[42] Singh B., Singh J.P., Kaur A., Singh N. (2020). Phenolic Composition, Antioxidant Potential and Health Benefits of Citrus Peel. Food Res. Int..

[43] Venkatakrishnan K., Chiu H.-F., Cheng J.-C., Chang Y.-H., Lu Y.-Y., Han Y.-C., Shen Y.-C., Tsai K.-S., Wang C.-K. (2018). Comparative Studies on the Hypolipidemic, Antioxidant and Hepatoprotective Activities of Catechin-Enriched Green and Oolong Tea in a Double-Blind Clinical Trial. Food Funct..

[44] Costabile G., Vitale M., Luongo D., Naviglio D., Vetrani C., Ciciola P., Tura A., Castello F., Mena P., Del Rio D. (2019). Grape Pomace Polyphenols Improve Insulin Response to a Standard Meal in Healthy Individuals: A Pilot Study. Clin. Nutr..

[45] Crowe-White K.M., Evans L.W., Kuhnle G.G.C., Milenkovic D., Stote K., Wallace T., Handu D., Senkus K.E. (2022). Flavan-3-Ols and Cardiometabolic Health: First Ever Dietary Bioactive Guideline. Adv. Nutr..

[46] Laurentin A., Edwards C.A. (2003). A Microtiter Modification of the Anthrone-Sulfuric Acid Colorimetric Assay for Glucose-Based Carbohydrates. Anal. Biochem..

[47] Huang Y.-P., Paviani B., Fukagawa N.K., Phillips K.M., Barile D. (2023). Comprehensive Oligosaccharide Profiling of Commercial Almond Milk, Soy Milk, and Soy Flour. Food Chem..

[48] Barnum C.R., Paviani B., Couture G., Masarweh C., Chen Y., Huang Y.-P., Markel K., Mills D.A., Lebrilla C.B., Barile D. (2024). Engineered Plants Provide a Photosynthetic Platform for the Production of Diverse Human Milk Oligosaccharides. Nat. Food.

[49] Magalhães P.J., Vieira J.S., Gonçalves L.M., Pacheco J.G., Guido L.F., Barros A.A. (2010). Isolation of Phenolic Compounds from Hop Extracts Using Polyvinylpolypyrrolidone: Characterization by High-Performance Liquid Chromatography-Diode Array Detection-Electrospray Tandem Mass Spectrometry. J. Chromatogr. A.

[50] Sinrod A.J.G., Li X., Bhattacharya M., Paviani B., Wang S.C., Barile D. (2021). A Second Life for Wine Grapes: Discovering Potentially Bioactive Oligosaccharides and Phenolics in Chardonnay Marc and Its Processing Fractions. LWT.

[51] Peng H., Paviani B., Nitin N., Barile D. (2025). Comprehensive Carbohydrate Profiling by Mass Spectrometry for the Valorization of Pomegranate Side Streams. Food Chem..

[52] Xu G., Amicucci M.J., Cheng Z., Galermo A.G., Lebrilla C.B. (2017). Revisiting Monosaccharide Analysis—Quantitation of a Comprehensive Set of Monosaccharides Using Dynamic Multiple Reaction Monitoring. Analyst.

[53] Škerget M., Kotnik P., Hadolin M., Hraš A.R., Simonič M., Knez Ž. (2005). Phenols, Proanthocyanidins, Flavones and Flavonols in Some Plant Materials and Their Antioxidant Activities. Food Chem..

[54] Zhao H., Avena-Bustillos R.J., Wang S.C. (2022). Extraction, Purification and In Vitro Antioxidant Activity Evaluation of Phenolic Compounds in California Olive Pomace. Foods.

[55] Ji M., Li C., Li Q. (2015). Rapid Separation and Identification of Phenolics in Crude Red Grape Skin Extracts by High Performance Liquid Chromatography Coupled to Diode Array Detection and Tandem Mass Spectrometry. J. Chromatogr. A.

